# Global symmetries and $\mathcal{N}=2$ SUSY

**DOI:** 10.1007/s11005-017-0952-0

**Published:** 2017-03-13

**Authors:** Jock McOrist, Ilarion V. Melnikov, Brian Wecht

**Affiliations:** 1 Department of Mathematics, University of Surrey Guildford, Surrey, GU2 7XH UK; 2 Department of Physics and Astronomy, James Madison University, Harrisonburg, VA USA; 3 Centre for Research in String Theory, Queen Mary University of London, London, E1 4NS UK

**Keywords:** Supersymmetric super Yang–Mills theory, Quantum field theory, Representations of Lie algebras, Symmetry breaking, 81T60 Supersymmetric field theories, 81T13 Yang–Mills and other gauge theories

## Abstract

We prove that $\mathcal{N}=2$ theories that arise by taking *n* free hypermultiplets and gauging a subgroup of ${\text {Sp}}(n)$, the non-R global symmetry of the free theory, have a remaining global symmetry, which is a direct sum of unitary, symplectic, and special orthogonal factors. This implies that theories that have ${\text {SU}}(N)$ but not ${\text {U}}(N)$ global symmetries, such as Gaiotto’s $T_N$ theories, are not likely to arise as IR fixed points of RG flows from weakly coupled ${\mathcal{N}=2}$ gauge theories.

## Introduction

Classifying the different possible phases of quantum field theories has been a long-standing goal of high-energy theoretical physics, and understanding and constraining the symmetries that arise in particular realizations is a key tool in this effort. In some cases, such as in two dimensions, there has been a significant amount of progress in this direction, e.g. the known restriction of unitary conformal field theories (CFTs) with $c<1$ to the minimal models, where the chiral algebra essentially fixes the theories. In four dimensions, however, significantly less is known, even in the case of CFTs.

It has long been known that it is possible to engineer four-dimensional CFTs, which do not obviously have any free-field limit. An early class of examples are the $\mathcal{N}=2$ SCFTs found by Minahan and Nemeschansky [1, 2]. These theories have ${\text {E}}_{6,7,8}$ global symmetries and can be studied via the Seiberg and Witten [3, 4] curve and the powerful techniques available in $\mathcal{N}=2$ theories. Although much is known about these theories, including the dimensions of various operators, ’t Hooft anomalies, and even some chiral ring relations [5], there is no known way of directly constructing the theories via an asymptotically free UV theory.^1^ It is worth noting though that recently [9] constructed an $\mathcal{N}=1$ theory that in certain limits is enhanced to $\mathcal{N}=2$ realizes an un-gauged Minahan–Nemeschansky $E_6$ theory. Shortly after the discovery of Argyres–Seiberg duality, it was realized [10] that the Minahan–Nemeschansky CFTs are in fact special cases of a much broader class of $\mathcal{N}=2$ theories that come from wrapping M5-branes on a three-punctured sphere. The ${\text {E}}_6$ theory is a special case of Gaiotto’s [10] $T_N$ theories, and ${\text {E}}_{7,8}$ are special cases that emerge when allowing more general punctures on the sphere [11–13]. For all but a few very special cases, which are free theories, these theories do not have known UV Lagrangian descriptions. Needless to say, such a description could be of great use—for instance, one could apply powerful localization techniques to constrain and perhaps fix the chiral ring structure of a given theory. This leads to a natural question: are there theories for which we can rule out the existence of a useful Lagrangian formulation?^2^

Despite the lack of a Lagrangian description, it is still possible to do detailed calculations in these theories. This is because for many quantities of interest, knowing information about the global symmetries such as the leading behaviour of current two- and three-point OPEs is sufficient, and global symmetry currents are among the limited set of operators to which we have reliable access. Although useful in general, global symmetry information has proved particularly important for studying $\mathcal{N}=1$ generalizations of the $T_N$ theories, as in [14] and subsequent work. This brings up the general question of what sorts of constraints follow from the global symmetries of these theories.

In this work, we make the observation that these two questions, i.e. the constraints on possible symmetries and existence of a Lagrangian, have an interesting relation in the context of $\mathcal{N}=2$ gauge theories. We will show that some (non-R) global symmetries, such as the ${\text {SU}}(N)^3$ global symmetry possessed by Gaiotto’s $T_N$ theories, are not straightforwardly realized by asymptotically free $\mathcal{N}=2$ theories. The essence of our argument is that such ${\text {SU}}(N)$ symmetries are always accompanied by an additional ${\text {U}}(1)$, which enhances the symmetry to ${\text {U}}(N)$. Although we will not be able to completely rule out the possibility that the $T_N$ theory has a UV Lagrangian description, we will be able to place constraints on any gauge theory realization. We will discuss these constraints and their limitations further in Sect. 4.

The main result of our paper is a proof that the global symmetries of certain $\mathcal{N}=2$ gauge theories fall into a straightforward classification depending on the matter representation. Our starting point will be a theory of *n* free hypermultiplets, which has a non-R global symmetry group ${\text {Sp}}(n)$. We prove that after gauging a subalgebra $\mathfrak {g}$ of the global symmetry algebra $\mathfrak {sp}(n)$, the remaining global symmetry algebra is a direct sum of $\mathfrak {so}, \mathfrak {sp}$, and ${\mathfrak u}$ factors. In particular, we note that $\mathfrak {su}$ factors without accompanying ${\mathfrak u}(1)$’s do not appear. This classification is certainly known to some experts (see, for example, [7, 15]), but we are not aware of a general proof in the literature. Our aim is to provide such a proof and explore some of the consequences.

## Symmetries of free fields

It is instructive to first understand the global symmetry of a theory of *n* free hypermultiplets. In $\mathcal{N}=1$ superspace, a hypermultiplet consists of a chiral superfield *Q* with propagating component fields $(q,\psi )$, and a chiral superfield $\widetilde{Q}$ with components $(\tilde{q},\widetilde{\psi })$. Requiring $\mathcal{N}=2$ supersymmetry implies there is a ${\text {U}}(1)_{\text {R}} \times {\text {SU}}(2)_{\text {R}}$ R-symmetry, under which $(q,\tilde{q}^\dag )$ transform as a doublet under ${\text {SU}}(2)_{\text {R}}$, while the fermions are neutral. We parametrize the ${\text {SU}}(2)_{\text {R}}$ action on the bosons as

1 $$\begin{aligned} \begin{aligned} T_{R} : \begin{pmatrix} q \\ \tilde{q}\end{pmatrix} \mapsto \begin{pmatrix} a q + b \tilde{q}^\dag \\ -b q^\dag + a \tilde{q}\end{pmatrix},\quad |a|^2 + |b|^2 = 1. \end{aligned} \end{aligned}$$

In what follows we split the 2*n* chiral multiplets into a column vector *Q* and a row vector $\widetilde{Q}$ (with transpose $\widetilde{Q}^t$), so that the Lagrangian for *n* free hypermultiplets is

2 $$\begin{aligned} \mathcal{L}= \int d^4\theta ~ \mathbf{Q}^\dag \mathbf{Q},\quad \mathbf{Q}\equiv \begin{pmatrix} Q \\ \widetilde{Q}^{t} \end{pmatrix}. \end{aligned}$$

We want to identify global symmetries that commute with both $\mathcal{N}=1$ and ${\text {SU}}(2)_{\text {R}}$. The first requirement means that these global symmetries must act linearly on the superfields $\mathbf{Q}$:

3 $$\begin{aligned} T_\mathbf{M}: \mathbf{Q}\rightarrow \mathbf{M}\mathbf{Q},\quad T_\mathbf{M}: \begin{pmatrix} Q \\ \widetilde{Q}^{t} \end{pmatrix} \rightarrow \begin{pmatrix} M_1 &{} N_1 \\ N_2 &{} M_2 \end{pmatrix} \begin{pmatrix} Q \\ \widetilde{Q}^{t} \end{pmatrix}, \end{aligned}$$

where $\mathbf{M}$ satisfies $\mathbf{M}{} \mathbf{M}^\dag = {\mathbbm {1}}_{2n}$, i.e. $\mathbf{M}\in {\text {U}}(2n)$. Since the ${\text {SU}}(2)_{\text {R}}$ acts trivially on fermions, we just need to determine the set of $\mathbf{M}$ restricted to the bosons that commute with the ${\text {SU}}(2)_{\text {R}}$ action. Evaluating the composition of two arbitrary rotations on the chiral fields explicitly,

4 $$\begin{aligned} T_{R} T_{\mathbf{M}}&: \begin{pmatrix} q \\ \tilde{q}\end{pmatrix} \mapsto \begin{pmatrix} a\left( M_1 q + N_1 \tilde{q}^t\right) + b \left( N_2^*q^*+ M_2^*\tilde{q}^\dag \right) \\ -b\left( \tilde{q}^*N_1^\dag + q^\dag M_1^\dag \right) + a\left( q^t N_2^t + \tilde{q}M_2^t\right) \end{pmatrix}~, \nonumber \\ T_{\mathbf{M}} T_{R}&: \begin{pmatrix} q \\ \tilde{q}\end{pmatrix} \mapsto \begin{pmatrix} a\left( M_1 q + N_1 \tilde{q}^t\right) + b\left( M_1 \tilde{q}^\dag -N_1 q^*\right) \\ a\left( q^t N_2^t + \tilde{q}M_2^t\right) + b\left( \tilde{q}^*N_2^t - q^\dag M_2^t\right) \end{pmatrix}~, \end{aligned}$$

we see that $[T_\mathbf{M},T_R]=0$ if and only if

5 $$\begin{aligned} M_1 = M_2^*,\quad N_1 = - N_2^*~. \end{aligned}$$

Equivalently, $\mathbf{M}J \mathbf{M}^t = J$, where *J* is the symplectic structure

6 $$\begin{aligned} J = \begin{pmatrix} 0 &{} {\mathbbm {1}}_{n} \\ -{\mathbbm {1}}_{n} &{} 0 \end{pmatrix}. \end{aligned}$$

Hence, $\mathbf{M}\in {\text {U}}(2n)\cap {\text {Sp}}(2n,{\mathbb C}) \equiv {\text {Sp}}(n)$, the compact unitary symplectic group.^3^ We have uncovered the global symmetry group of *n* free hypermultiplets: ${\text {U}}(1)_{\text {R}}\times {\text {SU}}(2)_{\text {R}} \times {\text {Sp}}(n)$, with matter in the fundamental of ${\text {Sp}}(n)$, a pseudoreal representation.^4^

## Representation theory

In this section, we will characterize the global symmetry algebra of a weakly coupled Lagrangian $\mathcal{N}=2$ gauge theory. Starting with a free theory of *n* hypermultiplets, we gauge a semisimple subalgebra $\mathfrak {g}$ of the global symmetry algebra $\mathfrak {sp}(n)$ of the free theory. The global symmetry algebra ${{\mathfrak {C}_{\mathfrak {g}}}}$ is the commutant of $\mathfrak {g}$ in $\mathfrak {sp}(n)$, i.e.

7 $$\begin{aligned} {{\mathfrak {C}_{\mathfrak {g}}}}= \{ x \in \mathfrak {sp}(n) ~|~~ {[x,y]} = 0\quad \text {for all}~ y \in \mathfrak {g}\}~. \end{aligned}$$

This is also known as the centralizer of $\mathfrak {g}$ in $\mathfrak {sp}(n)$. We will prove the following theorem.

### Theorem 1

Let $\mathfrak {g}$ be a semisimple subalgebra of $\mathfrak {sp}(n)$. Then, the commutant subalgebra ${{\mathfrak {C}_{\mathfrak {g}}}}$ of $\mathfrak {g}$ in $\mathfrak {sp}(n)$ is of the form

$$\begin{aligned} {{\mathfrak {C}_{\mathfrak {g}}}}= \bigoplus _i \mathfrak {sp}(k_i) \oplus \bigoplus _p \mathfrak {so}(l_p) \oplus \bigoplus _q \mathfrak {u}(m_q), \end{aligned}$$

and the fundamental of $\mathfrak {sp}(n)$ decomposes under $\mathfrak {sp}(n) \supset \mathfrak {g}\oplus \, {{\mathfrak {C}_{\mathfrak {g}}}}$ as

$$\begin{aligned} {{\varvec{2n}}} = \bigoplus _i \left( {{\varvec{r^+_i}}},{{\varvec{2k_i}}}\right) \oplus \bigoplus _p \left( {{\varvec{r^-_p}}},{{\varvec{l_p}}}\right) \oplus \bigoplus _q \left[ \left( {{\varvec{r^c_q}}}, {{\varvec{m_q}}}\right) \oplus \left( {{\overline{\varvec{r^c_q}}}},{{\overline{\varvec{m_q}}}}\right) \right] , \end{aligned}$$

where ${{\varvec{r^+_i}}}$, ${{\varvec{r^-_p}}}$, ${{\varvec{r^c_q}}}$ are distinct irreducible representations of $\mathfrak {g}$ that are, respectively, real, pseudoreal, or complex, and ${{\varvec{2k_i}}}$, ${{\varvec{l_p}}}$, and ${{\varvec{m_q}}}$ denote the fundamental representations of the corresponding factors in ${{\mathfrak {C}_{\mathfrak {g}}}}$.

The result has a simple implication for the physics: if we gauge a semisimple $\mathfrak {g}\subset \mathfrak {sp}(n)$, then the global symmetry group will be a sum of classical Lie algebras acting on the different flavours in fundamental representations.

In Sect. 3.1, we will illustrate Theorem 1 for a simple $\mathfrak {g}$. In Sect. 3.2, we review the branching rule for pseudoreal representations when $\mathfrak {g}$ is semisimple, before turning to a proof of Theorem 1 in Sect. 3.3.

### A few familiar gaugings for a simple $\mathfrak {g}$

Before we turn to the general case where $\mathfrak {g}$ is a semisimple algebra, we will review some familiar cases of $\mathcal{N}=2$ SQCD with $\mathfrak {g}$ a simple Lie algebra of type $\mathfrak {su}(p)$, $\mathfrak {sp}(q)$, or $\mathfrak {so}(m)$ [15, 16]. This gauging is accomplished by considering the embeddings

8 $$\begin{aligned} \mathfrak {sp}(pm)&\supset \mathfrak {su}(p)\oplus \mathfrak {u}(m)~,&{{\varvec{2pm}}}&= ({{\varvec{p}}},{{\varvec{m}}})\oplus ({{\overline{\varvec{p}}}},{{\overline{\varvec{m}}}})~,\nonumber \\ \mathfrak {sp}(qm)&\supset \mathfrak {sp}(q) \oplus \mathfrak {so}(m)~,&{{\varvec{2qm}}}&= ({{\varvec{2q}}},{{\varvec{m}}}). \end{aligned}$$

It is straightforward to then construct embeddings for any simple $\mathfrak {g}\subset \mathfrak {sp}(n)$. Suppose ${{\varvec{r}}}$ is an irreducible representation (irrep) of $\mathfrak {g}$ of dimension *k*. Then, depending on whether ${{\varvec{r}}}$ is real, pseudoreal, or complex, there is an S-subalgebra embedding $\mathfrak {g}\subset \mathfrak {so}(k)$, $\mathfrak {g}\subset \mathfrak {sp}(k)$, or $\mathfrak {g}\subset \mathfrak {su}(k)$, respectively [17]. It is then a simple matter to use the embeddings in (8) to construct suitable gauge theories. For instance, to build a $\mathfrak {e}_6$ gauge theory with *s* hypermultiplets in the ${{\varvec{27}}}$, we need *s* conjugate multiplets in ${{\overline{\varvec{27}}}}$, and we use the embedding

9 $$\begin{aligned} \mathfrak {sp}(27 s)&\supset \mathfrak {su}(27) \oplus \mathfrak {u}(s) \supset \mathfrak {e}_6 \oplus \mathfrak {u}(s),&{{\varvec{54 s}}} = ({{\varvec{27}}},{{\varvec{s}}}) \oplus ({{\overline{\varvec{27}}}},{{\overline{\varvec{s}}}}). \end{aligned}$$

In all of these cases, the reality properties of various irreps play a key role in constructing the embedding. As we will see, this will also be the case more generally. Our strategy will rely on two simple facts:

1. the decomposition of ${{\varvec{2n}}}$ under $\mathfrak {sp}(n)\supset \mathfrak {g}\oplus \, {{\mathfrak {C}_{\mathfrak {g}}}}$ determines the decomposition of ${\text {adj}}\,\mathfrak {sp}(n) = {\text {Sym}}^2{{\varvec{2n}}}$;
2. ${{\varvec{2n}}}$ is usefully decomposed according to reality properties of irreps of $\mathfrak {h}$, a semisimple subalgebra of $\mathfrak {g}$.

### Decomposing pseudoreal representations for semisimple $\mathfrak {g}$

We continue our warm-up by reviewing the branching rules for a pseudoreal representation of a semisimple Lie algebra $\mathfrak {g}$, which we will need for the proof of Theorem 1. We begin by fixing some useful conventions and reviewing a few definitions and familiar facts from representation theory. We only work with compact Lie algebras, so that all representations may be taken to be unitary (i.e. to admit an invariant Hermitian metric) with anti-Hermitian generators. We will denote the generators in representation ${{\varvec{r}}}$ by $\mathcal{T}_{{{\varvec{r}}}}$. The complex conjugate generators $\mathcal{T}^*_{{{\varvec{r}}}}$ define the conjugate representation ${{\overline{\varvec{r}}}}$, i.e. $\mathcal{T}^*_{{{\varvec{r}}}} = \mathcal{T}_{{{\overline{\varvec{r}}}}}$. The standard definitions for real/pseudoreal/complex representations are then as follows [17–19]:

#### Property 1

Let ${{\varvec{r}}}$ be an irrep of $\mathfrak {g}$.

- ${{\varvec{r}}}$ is real if there is a choice of basis such that the generators are real: $\mathcal{T}^*_{{{\varvec{r}}}} = \mathcal{T}_{{{\varvec{r}}}}$.
- ${{\varvec{r}}}$ is pseudoreal if there is a choice of basis such that $\mathcal{T}^*_{{{\varvec{r}}}} = \mathcal{J}\mathcal{T}_{{{\varvec{r}}}} \mathcal{J}^{-1}$ for some non-unitary matrix $\mathcal{J}$; Schur’s lemma and properties of complex matrices imply that $\mathcal{J}$ is anti-symmetric and $\mathcal{J}^2 = -{\mathbbm {1}}$, i.e. $\mathcal{J}$ is a complex structure on ${{\varvec{r}}}$.
- ${{\varvec{r}}}$ is complex if $\mathcal{T}^*_{{{\varvec{r}}}}$ and $\mathcal{T}_{{{\varvec{r}}}}$ are not related by a similarity transformation. In this case, $\mathcal{T}^*_{{{\varvec{r}}}}$ define the conjugate representation ${{\overline{\varvec{r}}}}$, and ${{\overline{\varvec{r}}}}$ is not equivalent to ${{\varvec{r}}}$ by a change of basis.

Schur’s lemma assures that these are mutually exclusive possibilities, and there is an equivalent characterization of the possibilities in terms of bilinear invariants of ${{\varvec{r}}}$: an irrep ${{\varvec{r}}}$ admits at most one bilinear invariant, which must either be symmetric or skew-symmetric [17–19], and this correlates with the reality properties of ${{\varvec{r}}}$ as follows.

#### Property 2

Let ${{\varvec{r}}}$ be an irrep of $\mathfrak {g}$.

- ${{\varvec{r}}}$ is real if and only if it admits a symmetric bilinear invariant, i.e. ${\text {Sym}}^2{{\varvec{r}}} \supset {{\varvec{1}}}$; in the basis where $\mathcal{T}_{{{\varvec{r}}}}^*= \mathcal{T}_{{{\varvec{r}}}}$, the bilinear is simply the identity. If, in addition, ${{\varvec{r}}}$ is faithful 5 , then $\wedge ^2{{\varvec{r}}} \supset {\text {adj}}\,\mathfrak {g}$.
- ${{\varvec{r}}}$ is pseudoreal if and only if it admits a skew-symmetric bilinear invariant, i.e. $\wedge ^2{{\varvec{r}}} \supset {{\varvec{1}}}$; in the basis where $\mathcal{T}^*_{{{\varvec{r}}}} = -\mathcal{J}\mathcal{T}_{{{\varvec{r}}}} \mathcal{J}$, and $\mathcal{J}$ is a complex structure on ${{\varvec{r}}}$, the bilinear is $\mathcal{J}$. In this case $\dim {{{\varvec{r}}}}$ is necessarily even. If, in addition, ${{\varvec{r}}}$ is faithful, then ${\text {Sym}}^2{{\varvec{r}}} \supset {\text {adj}}\,\mathfrak {g}$.
- ${{\varvec{r}}}$ is complex if it is neither real or pseudoreal, in which case ${{\varvec{r}}}\otimes {{\overline{\varvec{r}}}} \supset {{\varvec{1}}}$. If, in addition, ${{\varvec{r}}}$ is faithful, then ${{\varvec{r}}}\otimes {{\overline{\varvec{r}}}} \supset {{\varvec{1}}} \oplus {\text {adj}}\,\mathfrak {g}$.

We denote real, pseudoreal, and complex representations of $\mathfrak {g}$ by ${{\varvec{r^+}}}$, ${{\varvec{r^-}}}$, and ${{\varvec{r^c}}}$

The conjugate representation ${{\overline{\varvec{r}}}}$ of a semisimple $\mathfrak {g}$ is related by a similarity transformation to ${{\varvec{r}}}$ if and only if ${{\varvec{r}}}$ is real or pseudoreal. We see from above that for any irrep ${{\varvec{r}}}$, ${{\varvec{r}}}\otimes {{\overline{\varvec{r}}}}\supset {{\varvec{1}}}$. In fact, using crossing symmetry, that is associativity of the tensor product, we have the following result [19, 20]:

#### Lemma 1

Given two irreps ${{\varvec{r_1}}}$ and ${{\varvec{r_2}}}$ of a semisimple Lie algebra $\mathfrak {g}$, ${{\varvec{r_1}}} \otimes {{\varvec{r_2}}} \supset {{\varvec{1}}}$ if and only if ${{\varvec{r_1}}} = {{\overline{\varvec{r_2}}}}$.

The more general statement of crossing symmetry is that if ${{\varvec{r_1}}}\otimes {{\varvec{r_2}}} \supset {{\varvec{r_3}}}$, then ${{\varvec{r_1}}}\otimes {{\overline{\varvec{r_3}}}}$ contains ${{\overline{\varvec{r_2}}}}$. Our result follows by setting ${{\varvec{r_3}}} = {{\varvec{1}}}$.

Having reviewed some basic terminology, we end this section with two results on the branching of pseudoreal representations.

#### Lemma 2

Let ${{\varvec{R}}}$ be a pseudoreal irrep of a semisimple Lie algebra $\mathfrak {g}$, and let $\mathfrak {h}$ be a semisimple subalgebra of $\mathfrak {g}$. Then

$$\begin{aligned} {{\varvec{R}}} = \bigoplus _i \left( {{\varvec{r^+_i}}}\oplus {{\varvec{r^+_i}}}\right) ^{\oplus k_i} \oplus \bigoplus _p \left( {{\varvec{r^-_p}}}\right) ^{\oplus l_p} \oplus \bigoplus _q \left( {{\varvec{r^c_q}}}\oplus {{\overline{\varvec{r^c_q}}}}\right) ^{\oplus m_q}, \end{aligned}$$

where ${{\varvec{r^+_i}}}$, ${{\varvec{r^-_p}}}$, and ${{\varvec{r^c_q}}}$ are distinct real, pseudoreal, and complex irreps of $\mathfrak {h}$.

#### Proof

We can decompose ${{\varvec{R}}}$ as

10 $$\begin{aligned} {{\varvec{R}}} = \bigoplus _i \left( {{\varvec{r^+_i}}}\right) ^{\oplus K_i} \oplus \bigoplus _p \left( {{\varvec{r^-_p}}}\right) ^{\oplus l_p} \oplus \bigoplus _Q \left( {{\varvec{r^c_Q}}}\right) ^{\oplus m_Q}, \end{aligned}$$

where ${{\varvec{r^+_i}}}$, ${{\varvec{r^-_p}}}$, and ${{\varvec{r^c_Q}}}$ are inequivalent irreps with $K_i$, $l_p$ and $m_Q$ their multiplicity. The generators $\mathcal{T}_{{{\varvec{R}}}}$ are block-diagonal with respect to the decomposition and satisfy

11 $$\begin{aligned} \mathcal{J}\mathcal{T}^*_{{{\varvec{R}}}} = \mathcal{T}_{{{\varvec{R}}}} \mathcal{J}. \end{aligned}$$

$\mathcal{J}$ must act block-diagonally on each block of inequivalent real or pseudoreal representations in the sum. Furthermore, since ${{\varvec{r^c_Q}}}$ is not conjugate to ${{\overline{\varvec{r^c_Q}}}}$, in order to match the two sides of (11), ${{\varvec{r^c_q}}}$ occurs in the decomposition only if ${{\overline{\varvec{r^c_q}}}}$ occurs as well. Hence,

12 $$\begin{aligned} {{\varvec{R}}} = \bigoplus _i \left( {{\varvec{r^+_i}}}\right) ^{\oplus K_i} \oplus \bigoplus _p \left( {{\varvec{r^-_p}}}\right) ^{l_p} \oplus \bigoplus _q \left( {{\varvec{r^c_q}}}\oplus {{\overline{\varvec{r^c_q}}}}\right) ^{\oplus m_q}. \end{aligned}$$

Consider the action of $\mathcal{J}$ on $({{\varvec{r^+_i}}})^{\oplus K_i}$, denoted by $\mathcal{J}_i$, and let $n = \dim {{\varvec{r^+_i}}}$ so that $\mathcal{J}_i$ is a $n K_i\times n K_i$ matrix. Reality of ${{\varvec{r^+_i}}}$ means its generators $t_i$ may be taken to be real. Let $A,B,C,D \in \{1,\ldots , K_i\}$ and let $E_{AB}$ be a $K_i\times K_i$ matrix with $(E_{AB})_{CD} =\delta _{AC} \delta _{BD}$. Without loss of generality, we can write

$$\begin{aligned} \mathcal{J}_i = \textstyle \sum _{A,B} E_{AB} \otimes \tau _{AB}, \end{aligned}$$

where $\tau _{AB}$ are arbitrary $n\times n$ matrices acting on ${{\varvec{r^+_i}}}$. Therefore, the restriction of the requirement (11) to the block $({{\varvec{r^+_i}}})^{\oplus K_i}$ is

13 $$\begin{aligned} \sum _{A,B} E_{AB} \otimes \left( -t_i \tau _{AB} + \tau _{AB} t_i \right) = 0. \end{aligned}$$

Using the form of $E_{AB}$, this is only possible if ${[\tau _{AB},t_i]} = 0$ for all *A*, *B* and $t_i$. But, because ${{\varvec{r^+_i}}}$ is an irrep, that is only possible if $\tau _{AB} = x_{AB} {\mathbbm {1}}_{n}$ for some constants $x_{AB}$. Thus, $\mathcal{J}_i = M \otimes {\mathbbm {1}}_{n}$ for some $K_i\times K_i$ matrix *M*, and for $\mathcal{J}_i$ to be a complex structure, *M* must be skew-symmetric and satisfy $M^2 = -{\mathbbm {1}}_{K_i}$. Hence, $K_i = 2k_i$, and *M* is a complex structure on ${\mathbb C}^{k_i}$. So, the claim follows for the $({{\varvec{r^+_i}}})^{\oplus K_i}$ block.

Analogous considerations determine the action of the complex structure $\mathcal{J}$ on the remaining blocks: $\mathcal{J}_p = {\mathbbm {1}}_{l_p} \otimes j_p$, where $j_p$ is the bilinear invariant of ${{\varvec{r^-_p}}}$, while the action of $\mathcal{J}_q$ on $({{\varvec{r^c_q}}} \oplus {{\overline{\varvec{r^c_q}}}})^{\oplus m_q}$ has the same form as $\mathcal{J}_i$, but with $k_i$ replaced by $m_q$. The result follows. $\square$

We can decompose the previous result further with respect to $\mathfrak {h}\oplus \mathfrak {h}^{\prime }$, a semisible subalgebra of $\mathfrak {g}$.

#### Lemma 3

Let ${{\varvec{R}}}$ be a pseudoreal irrep of a semisimple Lie algebra $\mathfrak {g}$, and let $\mathfrak {h}\oplus \mathfrak {h}^{\prime }$ be a semisimple subalgebra of $\mathfrak {g}$. Decomposing ${{\varvec{R}}}$ with respect to $\mathfrak {h}\oplus \mathfrak {h}^{\prime }$, Lemma 2 is refined to

$$\begin{aligned} {{\varvec{R}}} = \bigoplus _i \left( {{\varvec{r^+_i}}},{{\varvec{R_i}}}\right) \oplus \bigoplus _p \left( {{\varvec{r^-_p}}},{{\varvec{R_p}}}\right) \oplus \bigoplus _q \left[ \left( {{\varvec{r^c_q}}},{{\varvec{R_q}}}\right) \oplus \left( {{\overline{\varvec{r^c_q}}}},{{\overline{\varvec{R_q}}}}\right) \right] . \end{aligned}$$

While ${{\varvec{R_i}}}$, ${{\varvec{R_p}}}$, ${{\varvec{R_q}}}$ need not be irreps of $\mathfrak {h}^{\prime }$ , $\wedge ^2{{\varvec{R_i}}}\supset {{\varvec{1}}}$ and ${\text {Sym}}^2{{\varvec{R_p}}}\supset {{\varvec{1}}}$.

The proof is simple: for instance, ${{\varvec{R_i}}}$ must admit a skew-symmetric invariant that plays the role of the matrix *M* in the proof of Lemma 2.

### Global symmetries

We now have the tools to prove Theorem 1, and we present the proof in this section. Let $\mathfrak {g}\subset \mathfrak {sp}(n)$ be a semisimple subalgebra with commutant ${{\mathfrak {C}_{\mathfrak {g}}}}$. It is easy to show that $\mathfrak {g}\cap \,{{\mathfrak {C}_{\mathfrak {g}}}}= 0$, so that $\mathfrak {g}\oplus \,{{\mathfrak {C}_{\mathfrak {g}}}}$ is a subalgebra of $\mathfrak {sp}(n)$, and ${{\mathfrak {C}_{\mathfrak {g}}}}$ is reductive, i.e. a sum ${{\mathfrak {C}_{\mathfrak {g}}}}= \mathfrak {h}\oplus \mathfrak {u}(1)^{\oplus A}$ of a semisimple factor $\mathfrak {h}$ and an abelian factor. Using Lemma 3, we decompose ${{\varvec{2n}}}$ as

14 $$\begin{aligned} {{\varvec{2n}}} = \bigoplus _{i} \left( {{\varvec{r^+_i}}}, {{\varvec{R_i}}}\right) \oplus \bigoplus _{p} \left( {{\varvec{r^-_p}}}, {{\varvec{R_p}}}\right) \oplus \bigoplus _{q} \left( {{\varvec{r^c_q}}},{{\varvec{R_q}}}\right) \oplus \left( {{\overline{\varvec{r^c_q}}}},{{\overline{\varvec{R_q}}}}\right) , \end{aligned}$$

where ${{\varvec{r^+_i}}}$, ${{\varvec{r^-_p}}}$ and ${{\varvec{r^c_q}}}$ denote distinct irreps of $\mathfrak {g}$ with indicated reality properties. Since ${\text {adj}}\,\mathfrak {sp}(n) = {\text {Sym}}^2{{\varvec{2n}}}$, we find

15 $$\begin{aligned} {\text {adj}}\,\mathfrak {sp}(n)&\supset \bigoplus _{i} \left( {\text {Sym}}^2{{\varvec{r^+_i}}}, {\text {Sym}}^2{{\varvec{R_i}}}\right) \oplus \bigoplus _p \left( \wedge ^2 {{\varvec{r^-_p}}}, \wedge ^2{{\varvec{R_p}}}\right) \nonumber \\&\quad \ \oplus \bigoplus _q \left( {{\varvec{r^c_q}}}\otimes {{\overline{\varvec{r^c_q}}}}, {{\varvec{R_q}}}\otimes {{\overline{\varvec{R_q}}}}\right) \nonumber \\&\supset \bigoplus _{i} \left( {{\varvec{1}}}, {\text {Sym}}^2{{\varvec{R_i}}}\right) \oplus \bigoplus _p \left( {{\varvec{1}}}, \wedge ^2{{\varvec{R_p}}}\right) \oplus \bigoplus _q \left( {{\varvec{1}}}, {{\varvec{R_q}}}\otimes {{\overline{\varvec{R_q}}}}\right) . \end{aligned}$$

By Lemma 1 every $\mathfrak {g}$-singlet in ${\text {adj}}\,\mathfrak {sp}(n)$ is obtained this way, and by assumption, these $\mathfrak {g}$ singlets are precisely the generators of ${{\mathfrak {C}_{\mathfrak {g}}}}$. Decomposing further into irreps of $\mathfrak {h}$ as

16 $$\begin{aligned} {{\varvec{R_i}}}&= \bigoplus _\alpha {{\varvec{\rho _{i\alpha }}}},&{{\varvec{R_p}}}&= \bigoplus _\sigma {{\varvec{\rho _{p\sigma }}}},&{{\varvec{R_q}}}&= \bigoplus _\mu {{\varvec{\rho _{q\mu }}}}, \end{aligned}$$

we obtain

17 $$\begin{aligned} {\text {adj}}\,\mathfrak {h}\oplus \mathfrak {u}(1)^A&= \bigoplus _{i} \bigoplus _{\alpha } {\text {Sym}}^2{{\varvec{\rho _{i\alpha } }}} \oplus \bigoplus _{p} \bigoplus _\sigma \wedge ^2 {{\varvec{\rho _{p\sigma }}}} \oplus \bigoplus _{q} \bigoplus _{\mu } {{\varvec{\rho _{q\mu }}}}\otimes {{\overline{\varvec{\rho _{q\mu }}}}} \nonumber \\&\quad \oplus \bigoplus _{i} \bigoplus _{\alpha>\beta } {{\varvec{ \rho _{i\alpha }}}}\otimes {{\varvec{\rho _{i\beta }}}} \oplus \bigoplus _{p} \bigoplus _{\sigma >\tau } {{\varvec{\rho _{p\sigma }}}}\otimes {{\varvec{\rho _{p\tau }}}} \oplus \bigoplus _{q} \bigoplus _{\mu \ne \nu } {{\varvec{\rho _{q\mu }}}}\otimes {{\overline{\varvec{\rho _{q\nu }}}}}. \end{aligned}$$

Decomposing $\mathfrak {h}= \oplus _s \mathfrak {h}_s$ into its simple summands, we observe that every summand in

18 $$\begin{aligned} {\text {adj}}\,\mathfrak {h}= \left( {\text {adj}}\,\mathfrak {h}_1,{{\varvec{1}}},\ldots ,{{\varvec{1}}}\right) \oplus \left( {{\varvec{1}}},{\text {adj}}\,\mathfrak {h}_2,\ldots ,{{\varvec{1}}}\right) \oplus \cdots \oplus \left( {{\varvec{1}}},\ldots ,{{\varvec{1}}},{\text {adj}}\,\mathfrak {h}_k\right) \end{aligned}$$

must be contained in exactly one of the summands in (17), and moreover, (17) cannot contain non-trivial representations of $\mathfrak {h}_s$ other than those appearing in (18). This implies that the second line of (17) must be absent; if it is present and contains the representations appearing in (18), then the first line of (17) will necessarily contain additional representations. This means that ${{\varvec{R_i}}}$, ${{\varvec{R_p}}}$ and ${{\varvec{R_q}}}$ must in fact be irreps of $\mathfrak {h}$.

Analogous reasoning show that each simple factor $\mathfrak {h}_s$ must act non-trivially on exactly one of ${{\varvec{R_i}}}$, ${{\varvec{R_p}}}$, ${{\varvec{R_q}}}$; otherwise, (17) will have additional non-trivial representations not contained in (18). So, we may write

19 $$\begin{aligned} \bigoplus _s \mathfrak {h}_s = \bigoplus _i \mathfrak {h}_i \oplus \bigoplus _p \mathfrak {h}_p \oplus \bigoplus _q \mathfrak {h}_q, \end{aligned}$$

with

20 $$\begin{aligned} {\text {adj}}\,\mathfrak {h}_i&= {\text {Sym}}^2{{\varvec{R_i}}},&{\text {adj}}\,\mathfrak {h}_p&= \wedge ^2{{\varvec{R_p}}},&\mathfrak {u}(1)^{\oplus A} \oplus \bigoplus _q {\text {adj}}\,\mathfrak {h}_q&= \bigoplus _q {{\varvec{R_q}}}\otimes {{\overline{\varvec{R_q}}}}. \end{aligned}$$

We recognize the classical groups $\mathfrak {h}_i = \mathfrak {sp}(k_i)$, $\mathfrak {h}_p = \mathfrak {so}(l_p)$, and $\mathfrak {h}_q = \mathfrak {su}(m_q)$, with ${{\varvec{R_i}}}$, ${{\varvec{R_p}}}$, and ${{\varvec{R_q}}}$ the corresponding fundamental representations. Moreover, the abelian factor $\mathfrak {u}(1)^{\oplus A} = \oplus _q \mathfrak {u}(1)_q$, and $\mathfrak {u}(1)_q$ acts with charge $+1$ on ${{\varvec{R_q}}}$ and $-1$ on ${{\overline{\varvec{R_q}}}}$. This completes the proof of Theorem 1.

## Discussion

Having found that gauging a subalgebra of $\mathfrak {sp}(n)$ does not yield $\mathfrak {su}(m)$ factors without accompanying ${\mathfrak u}(1)$’s, we now turn to the question of whether it is possible to get such factors in some other way. In particular, we consider two possibilities: gauging discrete subgroups and moving out on the Higgs branch. We will find that discrete gaugings do not yield $\mathfrak {su}(m)$’s, whereas special loci on the baryonic branch of SQCD do. Of course, we also cannot rule out the possibilities of emergent (accidental) symmetries yielding $\mathfrak {su}(m)$ factors, and we will have nothing further to say about this possibility here.

### Discrete gauge symmetries

One way to decrease the global symmetry group *G* is to introduce a further gauging by a discrete subgroup $\Gamma \subset G$. As shown in a beautiful paper [21], for $\mathcal{N}=2$ supersymmetric theories the only consistent gauging of global symmetries are combinations of the outer automorphism group of the flavour symmetry algebra, discrete subgroups of the $\mathrm{U}(1)_R$ and the low-energy EM duality group ${\text {SL}}(2,\mathbb {Z})$. The duality group contains the field theory coupling, and its gauging renders the theory non-perturbative. This is beyond the scope of our analysis here which is perturbative in nature.

### Higgs branch

The moduli space of $\mathcal{N}=2$ ${\text {SU}}(N_c)$ SQCD with $N_f$ flavours was comprehensively analysed in [15]. In this work, the authors describe the remaining global symmetries on the various possible sub-branches of the Higgs branch. When $N_c \le N_f < 2N_c$, the remaining non-R global symmetry on the baryonic branch is ${\text {SU}}(2N_c - N_f) \times {\text {U}}(1)^{N_f - N_c}$. When $N_f = N_c$, the ${\text {U}}(1)$ factors are spontaneously broken, and the global symmetry is simply ${\text {SU}}(N_f)$. Moreover, even when $N_f > N_c$, the ${\text {U}}(1)$ factors do not enhance ${\text {SU}}(2N_c - N_f)$ to ${\text {U}}(2N_c - N_f)$. Thus, it is possible to get non-enhanced ${\text {SU}}(m)$ factors on the Higgs branch of $\mathcal{N}=2$ theories.

### General discussion and conclusions

Let us now comment on some special cases of interest, in particular those of the low-rank $T_N$ theories. The first non-trivial case is the $T_2$ theory. This has a naïve global symmetry algebra $\mathfrak {su}(2)^{\oplus 3}$ and is known to be equivalent to a free theory of 8 chiral multiplets transforming in the tri-fundamental representation of $\mathfrak {su}(2)^{\oplus 3}$. From the perspective of the analysis in Sect. 2, it is clear that the global symmetry algebra is $\mathfrak {sp}(4)$, and under $\mathfrak {sp}(4) \supset \mathfrak {su}(2)^{\oplus 3}$ the matter decomposes as ${{\varvec{8}}} = ({{\varvec{2}}},{{\varvec{2}}},{{\varvec{2}}})$.

The $T_3$ theory has a similar structure. Naïvely this theory has a global symmetry algebra $\mathfrak {su}(3)^{\oplus 3}$ with chiral multiplets transforming in the tri-fundamental $({{\varvec{3}}},{{\varvec{3}}},{{\varvec{3}}})$. In fact, it is enhanced to ${\mathfrak e}_6$ [10], and the representation theory works out nicely: there is a maximal embedding $\mathfrak {su}(3)^{\oplus 3}\subset \mathfrak {e}_6$ under which

21 $$\begin{aligned} {{\varvec{78}}} = ({{\varvec{8}}},{{\varvec{1}}},{{\varvec{1}}})\oplus ({{\varvec{1}}},{{\varvec{8}}},{{\varvec{1}}}) \oplus ({{\varvec{1}}},{{\varvec{1}}},{{\varvec{8}}})\oplus ({{\varvec{3}}},{{\varvec{3}}},{{\varvec{3}}})\oplus ({{\overline{\varvec{3}}}},{{\overline{\varvec{3}}}},{{\overline{\varvec{3}}}})~. \end{aligned}$$

In other words, the tri-fundamental fields are additional global currents that enhance the naive $\mathfrak {su}(3)^{\oplus 3}$ to $\mathfrak {e}_6$.

Finally, consider the $T_4$ theory with its global symmetry algebra $\mathfrak {su}(4)^{\oplus 3}\cong \mathfrak {so}(6)^{\oplus 3}$ and matter in $({{\varvec{4}}},{{\varvec{4}}},{{\varvec{4}}})$. At first glance, one might hope that here a simple weakly coupled UV Lagrangian is not ruled out by our results, since of course we can easily construct an $\mathfrak {so}(6)^{\oplus 3}$ symmetry algebra. Alas, the hope is short-lived—in a theory so obtained the matter would transform in ${{\varvec{6}}}$ for each of the $\mathfrak {so}(6)$ factors, and no tensor product could produce the desired ${{\varvec{4}}}$ spinor representations.

We now conclude with a few brief comments. Although it is too strong to say that we have proven that $T_N$ theories do not arise via gauging the symmetries of free hypermultiplets, we have ruled out the simplest realizations that do not explore the Higgs branch of the $\mathcal{N}=2$ gauge theory. Consider moving out onto the Higgs branch by giving a field a vev *v*, and let the strong coupling scale of the UV gauge theory be denoted by $\Lambda$. If $v \gg \Lambda$, the IR gauge-neutral degrees of freedom, whose vevs parametrize the flat directions, will decouple from the IR gauge sector. The symmetries of the IR gauge theory will then again be constrained by Theorem 1. If, on the other hand, $v\sim \Lambda$, then the dynamics is necessarily strongly coupled and outside of the domain of validity of our results.

Of course a Lagrangian realization for $T_N$ has long been suspected to be highly unlikely, in light of the poorly understood dynamics of the M5-brane origin of such theories; for example, the $N^3$ scaling of the number of degrees of freedom in these systems does not seem to have any obvious gauge theory realization. Moreover, the $T_N$ theories have no marginal deformations, so they do not seem to arise as SCFTs in the same way as $N_f = 2N_c$ gauge theories, which have an exactly marginal gauge coupling.

However, even aside from possible applications to strongly coupled theories, our main result indicates just how strongly constrained the global symmetries of $\mathcal{N}=2$ gauge theories are and will perhaps provide a useful step towards a classification of such theories. For instance, by combining our results with the recent work [22], it should be easy to give a comprehensive list of all possible symmetry algebras of conformal and asymptotically free theories. It would be interesting to extend that to include possible discrete gaugings. It may perhaps also be useful to extend our results to $\mathcal{N}=1$ theories as well, though there we expect important new complications from possible superpotential interactions.

## Footnotes

1 It is worth noting that these theories, albeit with certain global symmetries gauged, can be realized via Argyres and Seiberg [6] duality and generalizations [7]. However, much like in the case of Argyres and Douglas [8] theories, there is not a straightforward mapping between the weakly coupled degrees of freedom and those of the un-gauged ${\text {E}}_n$ theories.

2 By utility we mean that the connection between UV and IR physics is relatively simple, ideally without the complications of a strong coupling limit or accidental symmetries.

3 In these conventions ${\text {Sp}}(1) = {\text {SU}}(2)$.

4 As discussed in [4], at the level of groups this action is not completely disjoint from that of the Lorentz group, but that will not affect our analysis at the level of the algebra.

5 Let $V_{{{\varvec{r}}}}$ denote the vector space of the irrep ${{\varvec{r}}}$. A representation $\mu _{{{\varvec{r}}}}: \mathfrak {g}\rightarrow \mathfrak {gl}(V_{{{\varvec{r}}}})$ is faithful if $\mu _{{{\varvec{r}}}}$ has a trivial kernel.

## References

[1] Minahan JA, Nemeschansky D (1997). Superconformal fixed points with E(n) global symmetry. Nucl. Phys. B.

[2] Minahan JA, Nemeschansky D (1996). An $$N=2$$ superconformal fixed point with E(6) global symmetry. Nucl. Phys. B.

[3] Seiberg N, Witten E (1994). Electric-magnetic duality, monopole condensation, and confinement in $$N=2$$ supersymmetric Yang–Mills theory. Nucl. Phys. B.

[4] Seiberg N, Witten E (1994). Monopoles, duality and chiral symmetry breaking in $$N=2$$ supersymmetric QCD. Nucl. Phys. B.

[5] Gaiotto D, Neitzke A, Tachikawa Y (2010). Argyres–Seiberg duality and the Higgs branch. Commun. Math. Phys..

[6] Argyres PC, Seiberg N (2007). S-duality in $$N=2$$ supersymmetric gauge theories. JHEP.

[7] Argyres PC, Wittig JR (2008). Infinite coupling duals of $$N=2$$ gauge theories and new rank 1 superconformal field theories. JHEP.

[8] Argyres PC, Douglas MR (1995). New phenomena in SU(3) supersymmetric gauge theory. Nucl. Phys. B.

[9] Gadde A, Razamat SS, Willett B (2015). “Lagrangian” for a non-lagrangian field theory with $$\cal{N} =2$$ supersymmetry. Phys. Rev. Lett..

[10] Gaiotto D (2012). $$N=2$$ dualities. JHEP.

[11] Tachikawa Y (2009). Six-dimensional D(N) theory and four-dimensional SO-USp quivers. JHEP.

[12] Chacaltana O, Distler J (2010). Tinkertoys for Gaiotto duality. JHEP.

[13] Chacaltana, O., Distler, J.: Tinkertoys for the $$D_N$$ series. arXiv:1106.5410 [hep-th]

[14] Benini F, Tachikawa Y, Wecht B (2010). Sicilian gauge theories and $$N=1$$ dualities. JHEP.

[15] Argyres PC, Plesser MR, Seiberg N (1996). The moduli space of vacua of $$N=2$$ SUSY QCD and duality in $$N=1$$ SUSY QCD. Nucl. Phys. B.

[16] Argyres PC, Plesser MR, Shapere AD (1997). $$N=2$$ moduli spaces and $$N=1$$ dualities for SO(n(c)) and USp(2n(c)) superQCD. Nucl. Phys. B.

[17] Cahn R (1985). Semi-simple Lie Algebras and Their Representations.

[18] McKay WG, Patera J (1981). Tables of Dimensions, Indices, and Branching Rules for Representations of Simple Lie Algebras. Lecture Notes in Pure and Applied Mathematics.

[19] Slansky R (1981). Group theory for unified model building. Phys. Rep..

[20] Di Francesco P, Mathieu P, Senechal D (1997). Conformal Field Theory.

[21] Argyres, P.C., Martone, M.: 4d $$\cal{N}=2$$ theories with disconnected gauge groups. arXiv:1611.08602 [hep-th]

[22] Bhardwaj, L., Tachikawa, Y.: Classification of 4d $$N=2$$ gauge theories. arXiv:1309.5160 [hep-th]

